# Potential probiotic yeasts isolated from the fish gut protect zebrafish (*Danio rerio*) from a *Vibrio anguillarum* challenge

**DOI:** 10.3389/fmicb.2015.01093

**Published:** 2015-10-07

**Authors:** Mario Caruffo, Natalie Navarrete, Oscar Salgado, Angélica Díaz, Paulina López, Katherine García, Carmen G. Feijóo, Paola Navarrete

**Affiliations:** ^1^Laboratorio de Microbiología y Probióticos, Instituto de Nutrición y Tecnología de los Alimentos, Universidad de ChileSantiago, Chile; ^2^Instituto de Ciencias Biomédicas, Universidad Autónoma de ChileSantiago, Chile; ^3^Departamento de Ciencias Biológicas, Facultad de Ciencias Biológicas, Universidad Andrés BelloSantiago, Chile; ^4^Interdisciplinary Center for Aquaculture Research, ConcepciónChile

**Keywords:** probiotic, yeast, aquaculture, *V. anguillarum*, zebrafish model system

## Abstract

Due to the negative consequences associated with the use of antibiotics, researchers, and food producers have studied alternatives, such as probiotics, for the control of fish diseases. The probiotic properties of yeasts in aquaculture have been scarcely considered. The present study investigated the probiotic properties of local yeast strains for aquaculture application in the protection of bacterial diseases. Yeast strains (*n* = 15), previously isolated from the intestinal gut of healthy salmonids, yellowtail, and croaker, were evaluated for their protection of zebrafish larvae following a *Vibrio anguillarum* challenge. We developed an infection model on zebrafish larvae with *V. anguillarum*, observing rapid mortality (≥50%) 5 days post-immersion challenge. Infection of Tg*(Lyz:DsRed*)^nz50^ larvae with fluorescent-marked *V. anguillarum* showed the oro-intestinal as the natural route of infection concomitant with an inflammatory response of the larvae reflected by neutrophil migration outside the hematopoietic tissue. Thirteen of 15 strains increased the percentage of larvae survival after the *V. anguillarum* challenge, although no yeast showed *in vitro* anti-*V. anguillarum* activity. In a subset of yeasts, we explored yeast–larvae interactions using fluorescent yeast and evaluated larvae colonization by culture analysis. All fluorescent yeasts were located in the gastrointestinal tract until 5 days post-inoculation (dpi). Yeasts reached 10^3^ CFU/larvae at 0 dpi, although the persistence until 5 dpi of the viable yeast in the gut was different among the strains. These results reveal that some yeasts isolated from the gut of fish could be potential probiotics, reducing the mortality associated to *V. anguillarum* challenge, and suggest that gut colonization could be involved in the protective effect. Future studies should elucidate other mechanisms involved in yeast protection and verify the beneficial effects of probiotic use in commercial fish species.

## Introduction

Aquaculture is one of the fastest-growing food producing sectors, providing almost 50% of all fish for human consumption (FAO/WHO, 2014). Chilean aquaculture has become an important economic activity with the harvest of salmonids (*Salmo salar, Oncorhynchus kisutch*, and *Oncorhynchus mykiss*), and new fish species with high economic potential, such as the carnivorous species croaker (*Cilus gilberti*), and yellowtail (*Seriola lalandi*). However, in large-scale production aquaculture farms, fish are exposed to different microbial diseases that can result in severe economic losses. One of the most common approaches to control fish diseases has been the use of antibiotics. Nevertheless, extensive documentation regarding the selection of resistant microorganisms, the spreading of resistant genes among pathogenic/commensal bacteria, and the environmental impact (contamination of water and sediments) associated with the use of antibiotics has led to the search for new alternatives, including probiotics (Newaj-Fyzul et al., 2014).

Yeasts have several attributes for consideration as good probiotic candidates. Yeasts are not affected by anti-bacterial compounds, and contain various immunostimulant compounds (e.g., β-glucans, nucleic acids, and mannan oligosaccharides), which can explain, in part, the protective effect against pathogens (Li and Gatlin, 2006; Lokesh et al., 2012). Moreover, some strains have antagonistic activities against undesirable bacteria (Hatoum et al., 2012), can stimulate intestine maturation (Tovar et al., 2002), and modulate antioxidant enzyme in host fish (Tovar-Ramírez et al., 2010). Despite their multiple attributes, screening of probiotic properties of yeasts have been mainly focused on a few species: *Saccharomyces cerevisiae* and, to a lesser extent, *Debaryomyces hansenii* (Gatesoupe, 2007).

Yeasts have been shown to constitute an important part of the microbiota of the fish gut (Gatesoupe, 2007; Navarrete and Tovar-Ramírez, 2014). Recently, we identified different yeast species in the gut microbiota of healthy wild and reared croaker (*C. gilberti*), yellowtail (*S. lalandi*), and salmonids, which may have potential probiotic properties (Raggi et al., 2014). The screening of beneficial properties of these candidates in commercial fish is a difficult task because of the long growth period of these fish, as well as the lack of controlled experimental facilities. These difficulties have led researchers to find alternative fish models with shorter growth period.

Zebrafish has been widely used as a research model for human development and disease (Vacaru et al., 2014). Due to the evolutionary proximity between zebrafish and cultured fish species reflected in many aspects of their biological similarity, zebrafish has been suggested as a model to study genetic (Dahm and Geisler, 2006), nutritional, and comparative growth studies (Ulloa et al., 2011). More recently, it has been used as a model organism to study pathogen–host interactions of infectious diseases (Sullivan and Kim, 2008; Rendueles et al., 2012; Shan et al., 2015) and gut microbiota/probiotic–host interactions (Bates et al., 2007; Kanther and Rawls, 2010; Rendueles et al., 2012; Llewellyn et al., 2014).

The aim of this study was to determine the probiotic properties of yeast strains isolated from the digestive tract of commercial fishes (salmonids, yellowtail, and croaker), specifically the protective effect of a *Vibrio anguillarum* challenge in zebrafish (*Danio rerio*).

## Materials and Methods

### Zebrafish Strains and Maintenance

The zebrafish strains used in this study were Tab 5 (wild type, WT) and Tg (*Lyz:DsRed*)^nz50^ (Hall et al., 2007). Zebrafish were maintained and raised according to Hedrera et al. (2013). All embryos were collected by natural spawning, staged according to Kimmel et al. (1995) and raised at 28°C in sterile E3 medium (Winkler, 0.5% NaCl, 0.17 mM KCl, 0.33 mM CaCl_2_, 0.33 mM MgSO_4_, and 0.05% methylene blue, pH 7.0) in six well plates. To avoid waste accumulation and oxygen limitation, we replaced 75% of the E3 volume with sterile E3 medium daily. Manipulation of zebrafish larvae for stereoscope analysis was done in anaesthetized larvae with tricaine methanesulfonate (4%, MS-222, Sigma–Aldrich). When appropriate, zebrafish larvae were euthanized with an overdose of tricaine according to Rendueles et al. (2012).

### *Vibrio anguillarum* Growth Conditions

The *V. anguillarum* strain was previously isolated from Atlantic salmon (*S. salar*) affected by haemorrhagic septicaemia (vibriosis) from a fish farm in the tenth region of southern Chile. The strain was cultured in Trypticase Soy Broth (TSB, BBL) supplemented with 0.5% NaCl at 28°C by 24 h. To do *V. anguillarum* counting, a 10-fold serial dilution of the homogenized zebrafish larvae in sterile phosphate buffer saline (PBS, Winkler) was plated in vibrio-selective medium thiosulfate citrate bile sucrose (TCBS, Oxoid) and CHROMagar^TM^ Vibrio medium (CHROMagar). As *V. anguillarum* strain used in this study grew better in CHROMagar^TM^ Vibrio medium (data not shown), the *Vibrio* counts were calculated from this media. Finally, inoculation of zebrafish was performed with cultures of *V. anguillarum* in exponential phase obtained at 28°C for 4 h.

### Yeast Strains and Growth Conditions

This study included 15 yeast strains comprising 7 yeast species previously isolated and identified from the gut of healthy salmonids, croaker, and yellowtail (Raggi et al., 2014). The yeast species and strains were selected according to their origin, enzyme production, or dominance in the fish guts (**Table 1**). Yeasts were cultured in YPD broth (1% yeast extract, Difco; 1% peptone, Difco; and 1% glucose, Merck) or YPD agar (YPD broth with 1.4% agar, Difco) supplemented with 0.05% chloramphenicol (Winkler), at 28°C under aerobic conditions. Inoculation of zebrafish larvae were performed with exponential cultures of yeast obtained in YPD broth at 28°C for 24 h.

**Table 1:** Yeast strains included in this study.

Yeast species	Phylum	Inclusion criteria^a^	Strains^b^	Origin of the yeast strains
*Candida deformans*	Ascomycota	Dominant species of salmonids (R) and croaker (W) gut determined by polymerase chain reaction- temporal temperature gradient gel electrophoresis (PCR-TTGE)^d^. Cultivable yeast of salmonids (R), yellowtail (W), and croaker (W) gut	Cd153 Cd154	croaker (W) croaker (W)
*Saccharomyces cerevisiae*	Ascomycota	Dominant species of salmonids (R), yellowtail (R, W), and croaker (R, W) gut determined by PCR-TTGE. Cultivable species of salmonids (R), yellowtail (R), and croaker (R) gut	Sc86	yellowtail (R)
*Rhodotorula mucilaginosa*	Basidiomycota	Cultivable species of salmonids (R, W), yellowtail (R, W) and croaker (R, W) gut	Rm9-1 Rm238	*Salmo salar* (R)
*Debaryomyces hansenii*	Ascomycota	Dominant species of salmonids (R), yellowtail (R, W) and croaker (R) gut determined by PCR-TTGE. Cultivable species of salmonids (R, W), yellowtail (R), and croaker (R, W)	Dh64 Dh72 Dh97 Dh132	yellowtail (R) yellowtail (R) *Oncorhynchus. mykiss* (R) croaker (R)
*Debaryomyces hansenii*	Ascomycota	Cultivable yeast of rainbow trout gut from a Swedish facility	CBS8339	*Salmo gairdneri* (R)^c^
*Yarrowia lipolytica*	Ascomycota	Dominant yeast of salmonids (R) gut determined by PCR-TTGE. Cultivable yeast of salmonids (R), yellowtail (W), and croaker (W) gut	Yl242	yellowtail (W)
*Metschnikowia viticola*	Ascomycota	Dominant yeast of salmonids (W) and croaker (W) gut determined by PCR-TTGE. Cultivable yeast of salmonids (W) gut	Mv5 Mv15	*O. mykiss* (W) *O. mykiss* (W)
*Cryptococcus laurentii*	Basidiomycota	Cultivable species of salmonids (W), and yellowtail (R) gut determined by culture. α-galactosidase producer	Cl21	*O. mykiss* (W)
*Candida* sp.	Ascomycota	Cultivable species of salmonids (R, W), and yellowtail (R) gut determined by culture.	Csp9	*O. mykiss* (W)

*^a,b^All yeast strains, except CBS8339, were isolated from Chilean fish species (Raggi et al., 2014). (R), reared fish; (W), wild fish. Fish species: salmonids (*O. kisutch, S. salar*, and *O. mykiss*), croaker (*Cilus gilberti*), and yellowtail (*Seriola lalandi*).*

*^c^*D. hansenii* CBS8339 was isolated from the gut of *Salmo gairdneri* (Andlid et al., 1995).*

*^d^PCR-TTGE, polymerase chain reaction-temporal temperature gradient gel electrophoresis.*

### *Vibrio anguillarum* and Yeasts Labeling

*Vibrio anguillarum* and yeasts were labeled according to Tovar et al. (2002), with a previous homogenization of 5-([4,6-Dichlorotriazin-2-yl] amino) fluorescein hydrochloride (DTAF, Sigma–Aldrich) in dimethyl sulfoxide (DMSO, Sigma–Aldrich).

### Experimental Challenges with *V. anguillarum* and Neutrophil Migration

Sixty WT larvae, 5 days post-fertilization (dpf) were randomly distributed in three wells of a six-well sterile tissue culture plates (triplicate, 20 larvae/well). *V. anguillarum* cultures were pelleted, resuspended in sterile E3 + 0.5% NaCl and transferred to each well at a final concentration of 10^7^ UFC/ml. Larvae were challenged by immersion with *V. anguillarum* for 20 min at 28°C, washed in sterile E3 + 0.5% NaCl and monitored for 5 days post-challenge. Control larvae were inoculated with E3 + 0.5% NaCl. More NaCl was added to E3 medium, because the survival of the halophilic bacteria *V. anguillarum* was enhanced in E3-containing 1% NaCl (data not shown).

The survival rate was recorded daily and life expectancy was determined as previously reported by Rendueles et al. (2012). Life expectancy corresponds to the average survival days of the larvae. Briefly, the larvae viability was monitored on a daily basis and mortality recorded. Experiments were performed at least three times. To determine *V. anguillarum* concentration in recently dead and surviving zebrafish (Rendueles et al., 2012), larvae were individually homogenized in sterile PBS and serial dilutions were plated in CHROMagar^TM^ Vibrio medium, during the experiment.

The route of infection and the effect of *V. anguillarum* in neutrophils migration outside the caudal hematopoietic tissue of larvae was assessed by fluorescent stereoscope observation using WT and Tg(*Lyz:DsRed*)^nz50^ strains, respectively. Zebrafish larvae (*n* = 60) were challenged with fluorescent *V. anguillarum* as described above. After the inoculation and during 3 h, zebrafish larvae were observed in an Olympus SZX16 stereoscope (Olympus) with a MicroPublisher 5.0 RVT camera (QImaging).

### Evaluation of the Protective Effect of Yeasts to *V. anguillarum* Challenge

Sixty WT larvae of 4 dpf larvae were randomly distributed in three wells (triplicate, 20 larvae/well) in a six-well plate. The yeast strains were grown at 28°C until the initial exponential phase, and then pelleted, resuspended in E3 + 0.5% NaCl and transferred to each well at a final concentration of 5 × 10^6^ UFC/ml for 2 h at 28°C. At 5 dpf, larvae pre-inoculated with each yeast were challenged with *V. anguillarum* as described above. Two control groups were included: (i) larvae inoculated with each yeast at 4 dpf (*n* = 60), and (ii) larvae inoculated with *V. anguillarum* at 5 dpf (*n* = 60). The survival rate was recorded daily and life expectancy was determined as previously reported and described above. Each experiment was independently performed three times.

### Determination of the *in vitro* Anti-*V. aguillarum* Activity of the Yeast Strains

The *in vitro* antibacterial activity of each yeast against *V. anguillarum* was evaluated using the diffusion method. Overnight culture of *V. anguillarum* adjusted to 0.5 McFarland (Biomérieux) was used to inoculate the surface of trypticase soy agar (TSA, BBL) supplemented with 0.5% NaCl. In these plates, six wells were performed to put 100 μl of a 48-h culture of each yeast. Plates were incubated overnight at 30°C, and the inhibition zone was measured the following day. These assays were performed three times.

### Colonization Studies and Microscopic Observation of the Yeast–Zebrafish Interactions

Zebrafish larvae (*n* = 60) were inoculated with each yeast and incubated as described above. Each day, zebrafish larvae were individually homogenized in sterile PBS and plated in YEPD agar supplemented with 0.05% chloramphenicol (Winkler) to obtain the yeast count (UFC/larvae). The interaction between yeast strains and the zebrafish larvae was performed through the inoculation of 5 × 10^6^ UFC/ml DTAF (Sigma) fluorescent yeast for 20 min. Observation of the larvae was performed at 0 and 5 dpi in an SZX16 stereoscope (Olympus) with a MicroPublisher 5.0 RVT camera (QImaging). Each experiment was independently performed three times.

### Imaging and Statistical Analysis

Images were processed with Photoshop CS5.1 (Adobe), with pictures showing the representative effects of each treatment. Statistical analysis was performed using the GraphPad Prism 5 software (Graphpad Software, Inc). Differences in number of neutrophils in the defined area in larvae were analyzed by applying Student’s *t*-test. Survival data were analyzed using Kaplan–Meier and group differences were analyzed by the log-rank test, using the Bonferroni correction for multiple comparisons. Life expectancy (zebrafish survival days) was analyzed using one-way ANOVA, using Dunnett’s test for multiple comparisons. Differences between average *V. anguillarum* CFU concentration in dying and survivor larvae were analyzed by applying Student’s *t*-test. *P* < 0.05 was considered significant.

### Ethical Statement

This study was carried out in strict accordance with the recommendations in the “Guidelines for the care and use of fish in Research” and the “Canadian Council on Animal Care’s Guide to the Care and Use of Experimental Animals” (CCAC Guide, 1989). The protocol was approved by the Committee on the Ethics of Animal Experiments of INTA, University of Chile and FONDECYT (FONDECYT 11110414).

## Results

### Challenge of Zebrafish Larvae with *V. anguillarum*

**FIGURE 1:**
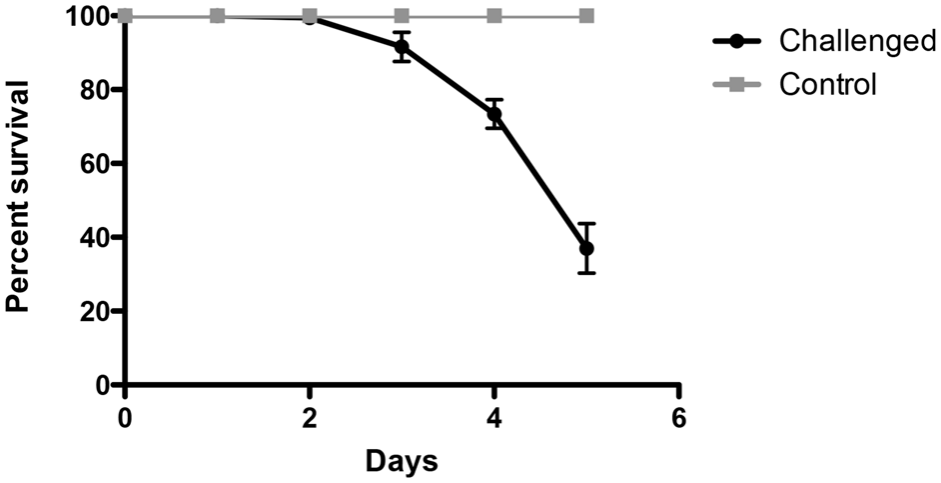
**Experimental challenge of zebrafish larvae with the fish bacterial pathogen *Vibrio anguillarum*.** 5 days post-fertilization (dpf) zebrafish larvae inoculated by immersion with 10^7^ UFC/ml *V. anguillarum* (*n* = 60). Comparison between challenged and control group showed significant differences in survival curves (Kaplan–Meier, Wilcoxon *P* < 0.0001).

**Figure 1** shows the survival curve of 5 dpf WT larvae exposed to *V. anguillarum*. Results show that *V. anguillarum* was highly virulent; for a period of 5 days post-challenge, survival of larvae challenged with *V. anguillarum* decreased to at least 45% compared to non-inoculated larvae (*P* < 0.0001). The average concentration of *V. anguillarum* in recently dead larvae was higher (4.0 ± 0.6 log_10_ CFU/larvae) than the average concentration of survivor larvae (2.0 ± 1.0 log_10_ CFU/larvae; *P* < 0.05).

**FIGURE 2:**
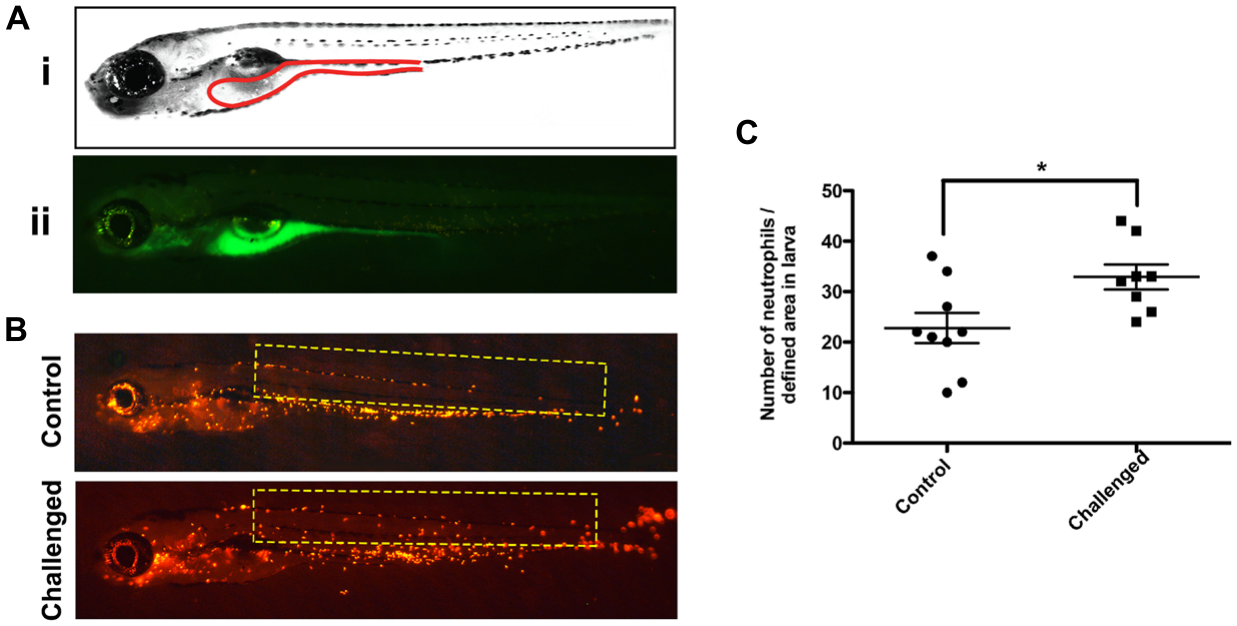
**Stereoscopic observation of the 5-([4,6-Dichlorotriazin-2-yl] amino) fluorescein hydrochloride (DTAF)-labeled *V. anguillarum* in zebrafish larvae. (A)** 5 dpf wild type (WT) larvae were inoculated by immersion with 10^7^ UFC/ml DTAF stained *V. anguillarum*: (i) lateral view scheme of the gut; (ii) lateral view of challenged larvae. **(B)** 5 dpf Tg*(Lyz:DsRed)^nz50^* larvae inoculated by immersion with 10^7^ UFC/ml DTAF-stained *V. anguillarum* (challenge) or sterile E3 as a control (control). **(C)** Quantification of neutrophils migration was performed in the selected area (yellow rectangle).

In order to assess the route of infection of *V. anguillarum* in the larvae, we labeled *V. anguillarum* with DTAF (Supplementary Figure S1). This labeling did not affect the viability of the bacteria checked by culturing them in CHROMagar^TM^ Vibrio medium (data not shown). Observation of WT larvae challenged with DTAF-*V. anguillarum* showed fluorescent bacteria in the gastrointestinal tract, and branchial arches from 1 to 3 h post-inoculation (**Figure 2A**) indicating that oral and gills were the main entry pathways of this pathogen. The infection of Tg(*Lyz:DsRed*)^nz50^ larvae with *V. anguillarum* induced mobilization of a high number of neutrophils outside the caudal hematopoietic tissue, in contrast to controls (*P* < 0.05), reflecting the stimulation of the innate immune system (**Figures 2B,C**).

### Screening of Protective Effect of Yeasts against *V. anguillarum* Infection in Zebrafish Larvae

**FIGURE 3:**
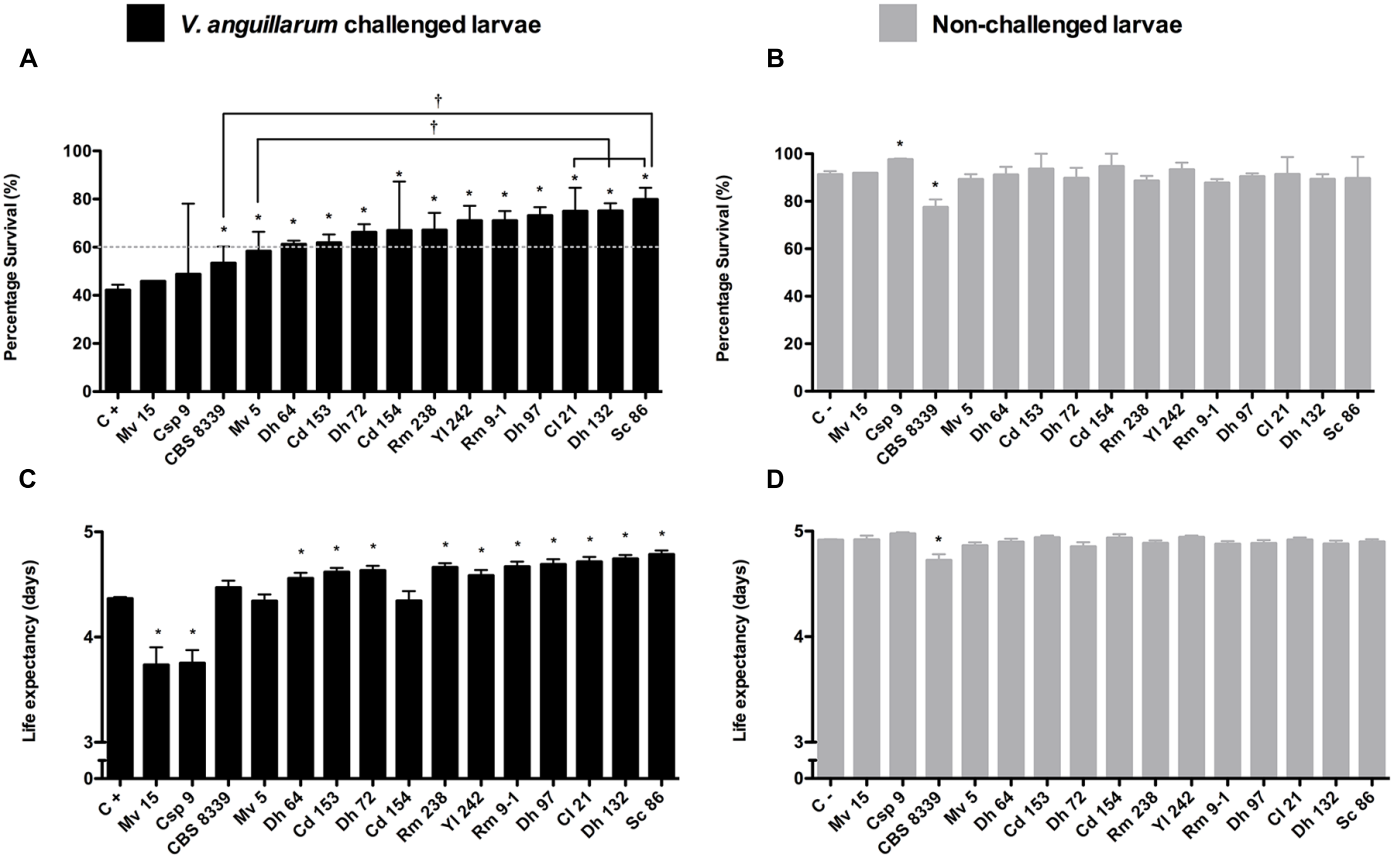
**Screening of protective effect of yeast against *V. anguillarum* challenge in zebrafish larvae.** 4 dpf larvae (*n* = 60) inoculated by immersion with 5 × 10^6^ UFC/ml yeast and challenged the 5 dpf by immersion with 10^7^ UFC/ml *V. anguillarum*. **(A,B)** Survival rate (%) at 6 days post-yeast inoculation. **(C,D)** Life expectancy (average of survival days of larvae). **(A,C)** Larvae inoculated with different yeasts and challenged with *V. anguillarum*; C+, control larvae challenged with *V. anguillarum.* **(B,D)** larvae inoculated with different yeast; C-, control larvae non-inoculated. The results show the average ±SD of three independent assays. Asterisks (^∗^) indicate significant differences with each respective control. Daggers (†) indicate significant differences among protective yeast.

The protective effect of 15 yeast strains (**Table 1**) was evaluated in the *V. anguillarum* infection model. The results show that 13 yeasts were able to protect larvae from the *V. anguillarum* challenge, significantly increasing rate survival (%; **Figure 3A**). Of these protective yeasts, those that increased average survival rate of challenged larvae to >60% were equally effective and did not show significant differences among them (**Figure 3A**). *D. hansenii* CBS8339 (CBS8339) and *Metschnikowia viticola* 15 (Mv5), which induced the lowest protection (<60%), showed significant differences with *Cryptococcus laurentii* 21 (Cl21), *D. hansenii* 132 (Dh132), and *S. cerevisiae* 86 (Sc86; **Figure 3A**). Of the 13 protective yeasts, 10 yeasts significantly increased the life expectancy of larvae (**Figure 3C**). The non-protective strains *M. viticola* 15 (Mv15) and *Candida* sp. 9 (Csp9; **Figure 3A**) significantly decreased the survival days of *V. anguillarum* challenged larvae (*P* < 0.001; **Figure 3C**).

Interestingly, Csp9 strain was the only yeast that significantly increased the survival rate of control non-*V. anguillarum* challenged larvae (*P* < 0.001; **Figure 3B**). Although the strain *D. hansenii* CBS8339 significantly increased the survival rate of challenged larvae (*P* < 0.05), the survival rate (**Figure 3B**), and life expectancy (**Figure 3D**) of yeast-inoculated larvae not challenged with *V. anguillarum* was significantly lower than the non-inoculated control (*P* < 0.0001).

### Colonization Assays of Yeast in Zebrafish Larvae

**FIGURE 4:**
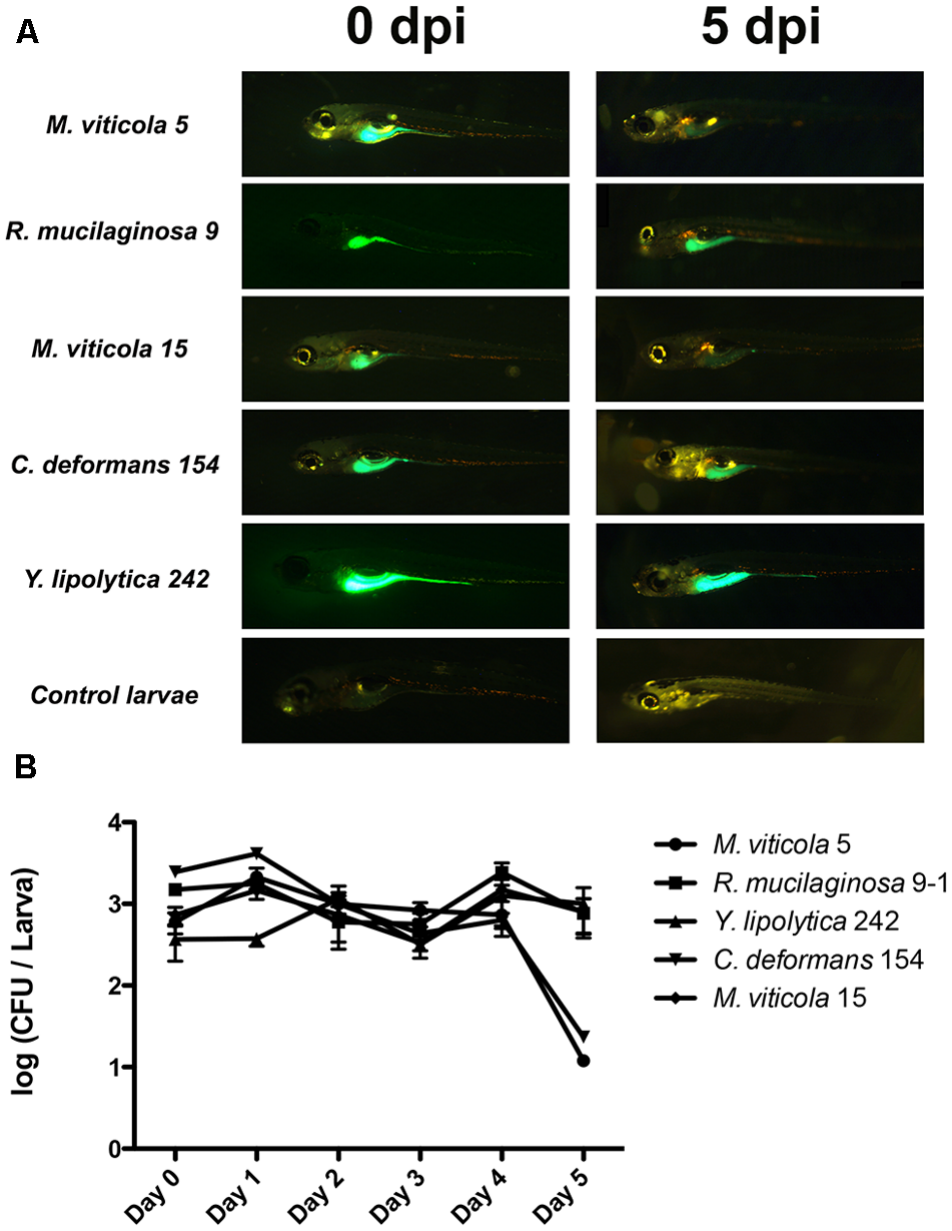
**Colonization studies and microscopic observation of the yeast–zebrafish interactions. (A)** Microscopic observation at 0 and 5 dpi of larvae (*n* = 60) inoculated by immersion with 5 × 10^6^ UFC/ml DTAF-fluorescent yeast. **(B)** Viable yeast count (UFC/larvae) of larvae inoculated by immersion with 5 × 10^6^ UFC/ml DTAF-fluorescent yeast.

For colonization studies, we selected four protective yeast strains isolated from different fish species (**Table 1**). Mv5 was isolated from *O. mykiss*, CBS8339 from *Salmo gairdneri, Candida deformans* 154 (Cd154) from *C. gilberti, Yarrowia lipolytica* 242 (Yl242) isolated from *S. lalandi*, and *Rhodotorula mucilaginosa* 9-1 (Rm9-1) from *S. salar*. Additionally, the non-protective yeast Mv15, isolated from *O. mykiss* was also evaluated. Fluorescent yeasts (Supplementary Figure S2) were predominantly observed in the digestive tract of larvae until 5 days post-yeast inoculation (**Figure 4A**). The viability of the yeasts in larvae revealed that at the day of inoculation (day 0), yeast counts reach 10^3^ CFU/larvae (**Figure 4B**), compared to non-inoculated larvae in which yeasts were not detected (<10 CFU/larvae). The persistence of viable yeasts in larvae was different among strains: *R. mucilaginosa* 9-1 (Rm9-1), *M. viticola* 15 (Mv15), and *Y. lipolytica* 242 (Yl242) were viable until 5 days post-inoculation (dpi), with yeast counts similar to those registered at the inoculation day. The counts of *M. viticola* 5 (Mv5) and *C. deformans* 154 (Cd154) started to decay at 4 dpi.

## Discussion

*Vibrio anguillarum* is a halophilic, Gram-negative bacterium and the causative agent of haemorrhagic septicaemia (vibriosis) which can affect several fish species including salt and freshwater fish (Toranzo et al., 2005; Frans et al., 2011) and can lead to important economic losses. In this study, we observed that the gastrointestinal tract and gills represented the principal entryways of *V. anguillarum*, as previously described in rainbow trout (Spanggaard et al., 2000) and zebrafish (O’Toole et al., 2004; Oyarbide et al., 2015). *V. anguillarum* counts in recently dead larvae were statistically higher than in survival larvae suggesting that the concentration of the pathogen in larvae could be related to the progression of the disease. This result is in agreement with a previous report showing that the number of the pathogen *Edwardsiella ictaluri* recovered from freshly euthanized homogenized infected zebrafish larvae increased daily until 3 days post-infection reaching a count similar to the one in our study shortly before death (Rendueles et al., 2012).

In the event of infection, neutrophils are the first immune cells to respond, and their role is to eliminate the pathogen through the production of anti-bacterial molecules such as lysozyme, cathepsins, and myeloperoxidase (MPO; van der Vaart et al., 2012). In this study, the use of the Tg(*Lyz:DsRed*)^nz50^ larvae allowed us to observed *in vivo* the potential of the pathogen *V. anguillarum* to mobilize labeled neutrophils as a reflection of the innate immune system stimulation (Medzhitov and Janeway, 2000). Using this model, we observed that the pathogen stimulated the innate immune system at 3 h post-*V. anguillarum* challenge, due to the high number of neutrophils mobilized outside the hematopoietic region. Neutrophil migration, as an innate immune activation marker, has also been observed in zebrafish in response to different *Flavobacterium psychrophilum* bath vaccines (Solís et al., 2015).

The use of probiotics, as an alternative to antibiotics, in aquaculture has been suggested to control bacterial diseases in fish (Balcázar et al., 2006; Kesarcodi-Watson et al., 2008). Some attributes of potential probiotics microorganisms for aquaculture have been suggested (Merrifield et al., 2010). To be better adapted to the gastrointestinal conditions, probiotic candidates should ideally be indigenous to the host (Merrifield et al., 2010). Thus, the different yeast species isolated from the gut of local fish (Raggi et al., 2014) are good probiotics candidates. Here, we tested the protective effect of 15 yeast isolates belonging to 7 different yeast species, including *Candida* sp., *R. mucilaginosa, Y. lipolytica, M. viticola, C. laurentii, D. hansenii*, and *S. cerevisiae.* All these isolates were recovered from healthy fish which suggests that they are non-pathogenic. The majority of the yeast isolates were safe since they did not affect the survival rate of the larvae. Interestingly, *Candida* sp. 9, increased the survival rate of the larvae; while *D. hansenii* CBS8339 decreased it. The improvement of fish survival rate by yeast supplementation, as we observed with *Candida* sp. 9, has been previously reported in European sea bass larvae (Tovar-Ramírez et al., 2004). This beneficial effect was described for the yeast strain *D. hansenii* CBS8339 (HF1), the same strain used in this study. The survival improvement by the CBS8339 strain has been associated with an early enterocyte maturation of the larvae stimulated by polyamines produced by the yeast (Tovar-Ramírez et al., 2004). The eventual polyamine production of *Candida* sp. 9 should be checked in future studies. In our study, CBS8339 strain showed opposite results, reducing the survival rate. This observation shows that the same strain may cause dissimilar effects in different hosts, highlighting the need to check yeast safety in the target fish species. In this regard, an important aspect to be analyzed would be the yeast viability in the aquatic medium to determine its potential environmental impact and eventually affect the survival of susceptible host.

Using the *V. anguillarum* infection model in zebrafish, we detected 13 yeasts that protected larvae against the *V. anguillarum* challenge, significantly increasing their survival rate compared to *V. anguillarum* challenged larvae (*P* < 0.05). Few studies determining the protective effect of yeasts against fish microbial pathogens have been reported, probably due to the extensive labor costs and time demands of these experiments. In previous studies, yeasts have showed protective effects against a broad range of pathogens, including parasites, bacteria, and virus. Harikrishnan et al. (2011) showed that baker’s yeast *S. cerevisiae* (KCCM 11201)-supplemented diets enhanced the survival rate of *Uronema marinum*-infected olive flounder (*Paralichthys olivaceus*). Groupers (*Epinephelus coioides*) fed a diet containing *S. cerevisiae* P13, isolated from fermented peaches, had significantly higher survival rates than the control diet after a challenge with *Streptococcus* sp. and an iridovirus, respectively (Chiu et al., 2010). On the other hand, *D. hansenii* (CBS8339) protected stressed juvenile leopard grouper (*Mycteroperca rosacea*) against the dinoflagellates *Amyloodinium ocellatum* infection (Reyes-Becerril et al., 2008).

Colonization experiments showed that yeasts were able to colonize and remain viable in the digestive system of larvae for at least 4 dpi. To our knowledge, this is the first report regarding this issue in zebrafish. Since colonization has been regarded has one of the desirable properties for probiotics (Balcázar et al., 2006), colonization capacities of some yeasts, measured as viable yeast in the digestive tract, have been demonstrated in sea bass (*Dicentrarchus labrax*) larvae (Tovar et al., 2002) and rainbow trout (*O. mykiss*) fry (Waché et al., 2006). However, in those studies fish were constantly fed yeast-supplemented diets; therefore, the persistence of a unique dose of these yeast strains in the fish gut is unknown. In this study, the persistence of one yeast administration in the larvae gut was higher than the intestinal transit time reported for zebrafish larvae (24 h; Field et al., 2009). The permanence of the yeast in zebrafish gut can be explained by their capacity to adhere and grow in intestinal mucus (Andlid et al., 1998).

We did not detect anti-*V. anguillarum* activity *in vitro*, suggesting that larvae protection could be explained by an antibacterial activity expressed only *in vivo* or by other mechanisms different from antimicrobial compound production (e.g., nutrient competition, pH modification, or ethanol production; Hatoum et al., 2012). The gut colonization capacity of the yeasts suggests that competitive exclusion mechanisms could be involved in protection. Furthermore, some cell wall components of the yeast (β-glucans, mannans, and chitin) could participate in yeast-*V. anguillarum* interaction, such as co-aggregation (Hatoum et al., 2012), reducing the pathogen entry to host through the digestive tract. On the other hand, yeast protection of fish from microbial pathogens, has also been related to the stimulation of the immune system of the host (Reyes-Becerril et al., 2008; Chiu et al., 2010; Harikrishnan et al., 2011). The specific mechanisms triggered by yeast involved in larvae protection, such as anti *in vivo V. anguillarum* activity, yeast–*V. anguillarum* co-aggregation or modulation of the immune system (neutrophil mobilization or cytokines expression) will be the objective of future studies. Elucidating mechanisms involved in larval protection of each yeast will also help to explain differences observed in protection capacity among the strains.

## Conclusion

We reported 13 potential probiotic yeast strains with protective activity against a *V. anguillarum* challenge, belonging to different yeast species including *S. cerevisiae* and *D. hansenii.* The probiotic screening of yeasts in the zebrafish model showed several experimental advantages: zebrafish larvae were easy to maintain, protection experiments were developed in short period of time, and yeast colonization could be observed *in vivo*, due to the optical clarity of larvae. This fish model may help to elucidate the mechanisms underlying the protective effect of yeast. Future studies will be performed to determine the beneficial effect of yeast strains analyzed in this work in commercial fish.

## Author Contributions

MC, NN, and PN conceived and designed the experiments. MC, NN, OS, AD, and PL performed the experiments. MC, NN, KG, CF, and PN analyzed the data. MC and PN wrote the paper.

## Conflict of Interest Statement

The authors declare that the research was conducted in the absence of any commercial or financial relationships that could be construed as a potential conflict of interest.
