# Supplementary material for: Potential probiotic yeasts isolated from the fish gut protect zebrafish (Danio rerio) from a Vibrio anguillarum challenge
Source: Front Microbiol. 2015 Oct 7;6:1093. doi: 10.3389/fmicb.2015.01093 (PMC4596066; doi:10.3389/fmicb.2015.01093)

Figure S1. DTAF-labeled *Vibrio anguillarum*. The bacteria was observed under fluorescence microscope (excitation 495 nm, emission 516 nm; 400X)

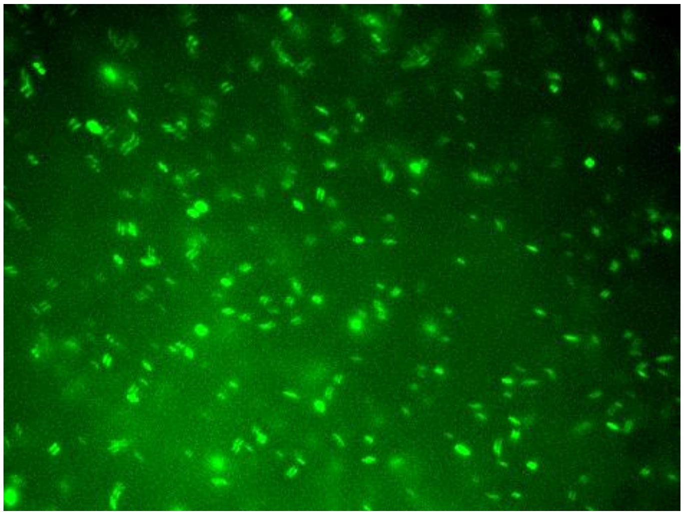

Supplement: Supplementary file 1 [file Image_1.PDF]
